# Hyperthermia restores apoptosis induced by death receptors through aggregation-induced c-FLIP cytosolic depletion

**DOI:** 10.1038/cddis.2015.12

**Published:** 2015-02-12

**Authors:** A Morlé, C Garrido, O Micheau

**Affiliations:** 1 INSERM, UMR866, Dijon, F-21079 France; 2 Faculty of Medicine and Pharmacy, Univ. Bourgogne, Dijon, F-21079 France; 3 Centre Georges-François Leclerc, Dijon, F-21000 France

## Abstract

TRAIL is involved in immune tumor surveillance and is considered a promising anti-cancer agent owing to its limited side effects on healthy cells. However, some cancer cells display resistance, or become resistant to TRAIL-induced cell death. Hyperthermia can enhance sensitivity to TRAIL-induced cell death in various resistant cancer cell lines, including lung, breast, colon or prostate carcinomas. Mild heat shock treatment has been proposed to restore Fas ligand or TRAIL-induced apoptosis through c-FLIP degradation or the mitochondrial pathway. We demonstrate here that neither the mitochondria nor c-FLIP degradation are required for TRAIL-induced cell death restoration during hyperthermia. Our data provide evidence that insolubilization of c-FLIP, alone, is sufficient to enhance apoptosis induced by death receptors. Hyperthermia induced c-FLIP depletion from the cytosolic fraction, without apparent degradation, thereby preventing c-FLIP recruitment to the TRAIL DISC and allowing efficient caspase-8 cleavage and apoptosis. Hyperthermia-induced c-FLIP depletion was independent of c-FLIP DED2 FL chain assembly motif or ubiquitination-mediated c-FLIP degradation, as assessed using c-FLIP point mutants on lysine 167 and 195 or threonine 166, a phosphorylation site known to regulate ubiquitination of c-FLIP. Rather, c-FLIP depletion was associated with aggregation, because addition of glycerol not only prevented the loss of c-FLIP from the cytosol but also enabled c-FLIP recruitment within the TRAIL DISC, thus inhibiting TRAIL-induced apoptosis during hyperthermia. Altogether our results demonstrate that c-FLIP is a thermosensitive protein whose targeting by hyperthermia allows restoration of apoptosis induced by TNF ligands, including TRAIL. Our findings suggest that combining TRAIL agonists with whole-body or localized hyperthermia may be an interesting approach in cancer therapy.

TRAIL holds promise in the clinic owing to its anti-tumoral selectivity.^1^ Evaluation of TRAIL in patients has, however, proved less efficient than anticipated.^2^ Among the regulatory mechanisms that may explain TRAIL resistance, c-FLIP, which is highly expressed in primary tumors and often associated with bad prognosis,^3, 4, 5^ is likely to play the most important role. Targeting c-FLIP has clearly emerged as an important issue for cancer therapies. So far, three isoforms have been described. The long isoform, c-FLIP_L_, composed of two death effector domains (DED) and a caspase-like domain devoid of the prototypic catalytic cysteine contained in pro-caspases,^6^ and two short isoforms, c-FLIP_S_ and c-FLIP_R,_ mainly composed of the two DEDs.^7^ Regardless of the isoform, c-FLIP proteins are co-recruited within the DISC of death domain (DD)-containing receptors of the TNF superfamily and prevent the release of active caspase-8 to the cytosol, inhibiting apoptosis induced by these death receptors.^8, 9^

Expression levels of c-FLIP proteins are regulated both transcriptionally and posttranscriptionally. At the transcriptional level, c-FLIP isoforms are repressed by transcription factors including E2F1 or c-Myc,^10, 11^ or induced by NF-kB.^12, 13^ Regulation of c-FLIP expression by NF-kB plays a central role in protecting cells from TNF-induced cell death.^14^ This pro-inflammatory signaling pathway contributes to sustained expression of c-FLIP in primary tumors and confers resistance to apoptosis induced by death receptors.^15, 16^ At the posttranslational level, c-FLIP proteins are regulated through the ubiquitin-proteasomal pathway. Ubiquitination of c-FLIP on lysines 167 or 195 induces its degradation by the proteasome.^17, 18^ Phosphorylation of c-FLIP can also lead to the regulation of c-FLIP ubiquitination and degradation. Activation of PKC induces c-FLIP phosphorylation on serine 193 and inhibits c-FLIP_s_ ubiquitination and degradation.^19^ ROS can also induce c-FLIP phosphorylation on threonine 166, leading to c-FLIP ubiquitination on lysine 167 and degradation by the proteasome.^18^ Several ubiquitin ligases contribute to c-FLIP ubiquitination including itch, c-Cbl and AIP4.^20, 21, 22^ Consistent with the increasing body of evidence demonstrating that a large number of stimuli lead to c-FLIP degradation and restoration of apoptosis induced by death receptors,^7^ hyperthermia has recently been proposed to restore TRAIL pro-apoptotic signaling through ubiquitination of c-FLIP on K195.^17^

Herein, we provide evidence that proteosomal-mediated degradation of c-FLIP, albeit induced during hyperthermia, is not required for sensitization or restoration of TRAIL-induced cell death. Instead, our findings demonstrate that both c-FLIP isoforms are thermolabile proteins that aggregate during hyperthermia. As a consequence, c-FLIP proteins are not available in the cytosol and their recruitment within the TRAIL DISC is impaired, which allows efficient initiator caspase activation.

## Results

### Hyperthermia restores TRAIL-induced apoptosis in a mitochondrial-independent manner

**Figure 1:**
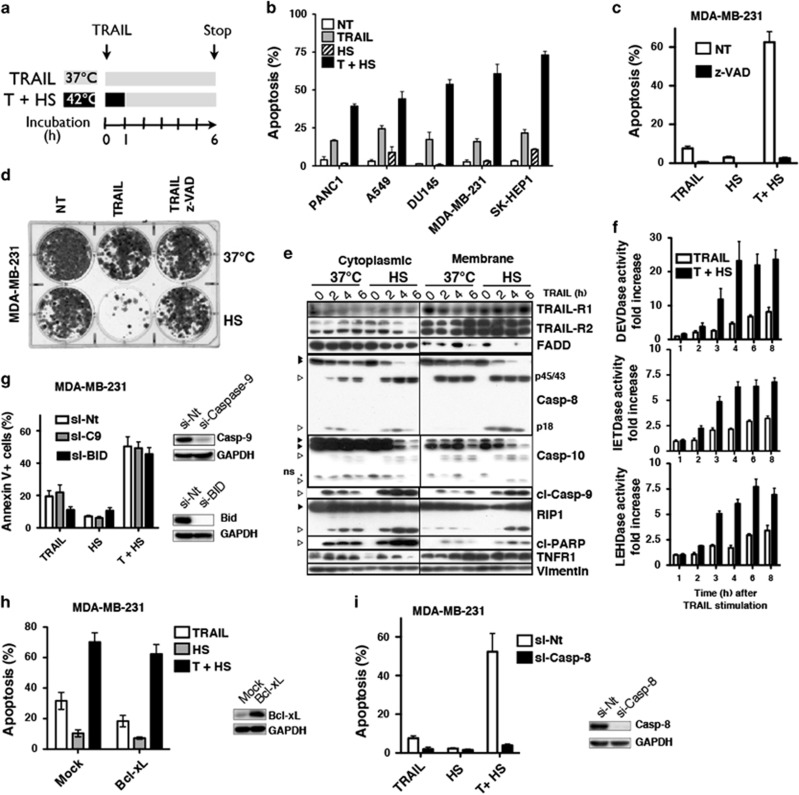
Hyperthermia increases TRAIL-induced cell death in a caspase-dependent but mitochondrial-independent manner. (**a**) Schematic representation of the protocol used to stimulate cells with TRAIL. Cells were either stimulated at 37 °C for 6 h, or incubated in the presence (T+HS) or absence (HS) of His-TRAIL for 1 h at 42 °C followed by a 5 h additional incubation at 37 °C. (**b**) Indicated cancer cell lines were stimulated with 500 ng/ml His-TRAIL (TRAIL) or TRAIL and hyperthermia (T+HS) as described above and apoptosis was measured after 6 h after the onset of the stimulation by Hoechst staining. (**c**) MDA-MB-231 cells were pre-incubated or not 30 min with 5 *μ*M caspase inhibitor z-VAD and stimulated with 50 ng/ml His-TRAIL. Apoptosis was measured after 6 h by Hoechst staining. (**d)** Five hundred MDA-MB-231 cells, plated overnight in a 6-well plate, were pre-incubated or not for 30 min with 20 *μ*M z-VAD prior stimulation or not with 500 ng/ml His-TRAIL at 37 °C or in hyperthermic condition (HS) for 1 h and allowed to recover for a week at 37 °C before staining with methylene blue. (**e**) MDA-MB-231 cells were stimulated or not with 500 ng/ml His-TRAIL as indicated for 2, 4 or 6 h and cytosolic or membrane fractions were isolated (see methods). Expression levels of indicated proteins were detected by immunoblotting. One representative blot is shown (*n*=3). (**f**) MDA-MB-231 cells were stimulated as described above with 50 ng/ml His-TRAIL and caspase activities were measured by luminometry 1–8 h after stimulation using caspase-3/7 (DEVD), caspase-8/10 (IETD) or caspase-9 (LEHD) luminogenic substrates. (**g**) MDA-MB-231 cells were transfected with non-targeting (si-Nt), caspase-9 (si-C9) or BID (si-BID) targeting siRNAs. Seventy-two hours after transfection, cells were stimulated or not with 100 ng/ml His-TRAIL and apoptosis was analyzed after 6 h by Annexin V staining and flow cytometry. Caspase-9 and BID expression levels are shown on the right. (**h**) MDA-MB-231 cells, transfected with an empty retroviral vector (Mock) or a vector encoding Bcl-xL, were stimulated as above and apoptosis induced by the indicated stimuli was analyzed by Hoechst. Bcl-xL expression is shown on the right. (**i**) MDA-MB-231 cells were transfected with a non-targeting (si-Nt) or caspase-8 (si-C8) targeting siRNA, stimulated or not 72 h after transfection with 100 ng/ml His-TRAIL and apoptosis was analyzed as above. Caspase-8 expression levels were controlled by immunoblotting. (**b**, **c**, **d**, **g**, **h** and **i**) Error bars represent S.D. from at least three independent experiments

Hyperthermia restores TRAIL-induced apoptosis in tumor cells^17, 23, 24^ but not in normal cells.^25^ In line with these findings, incubating resistant cancer cell lines of various origin for 1 h at 42 °C (HS) in the presence of TRAIL followed by subsequent incubation at 37 °C for 5 h (Figure 1a), significantly increased apoptosis triggered by TRAIL as compared with a 6 h incubation time at 37 °C (Figure 1b). Incubation of the cells during the first hour at milder temperature, that is 39 °C, or at 0 °C failed to do so (Supplementary Figures 1a and b). As expected, incubating cells for 1 h at 42 °C was sufficient to induce a time-dependent upregulation of inducible HSPs including HSP27, *α*B-crystallin, HSP70 or HSP110 (Supplementary Figure 1c), phosphorylation of HSP27 on serine 15, 78 and 82, translocation of HSP27 into the non-ionic detergent insoluble fraction and to increase protein ubiquitination (Supplementary Figure 1d). However, restoration of TRAIL pro-apoptotic activity by HS (Figure 1b) was not correlated with steady state differential expression levels of HSPs (Supplementary Figure 2b) or TRAIL receptors but to some extent with c-FLIP expression levels (Supplementary Figures 2a and b). Likewise, regardless of their initial sensitivity to TRAIL (Supplementary Figure 2c), cells expressing high amounts of c-FLIP were more responsive to TRAIL during HS (Figure 1b) than cells expressing low levels (Supplementary Figure 2).

To understand the molecular mechanisms underlying the gain of function during hyperthermia, we decided to use the resistant mammary carcinoma cell line MDA-MB-231 as a model cell line. Cell death induced by TRAIL after a HS, was mainly driven by caspases as the pan-caspase inhibitor z-VAD totally abrogated apoptosis induced by TRAIL (Figure 1c) and rescued MDA-MB-231 clonogenic growth after TRAIL stimulation (Figure 1d). As evidenced by immunoblotting, hyperthermia enhanced initiator caspase-8, -9 and -10 processing, as compared with control conditions (37 °C), both in the cytosolic and the membrane-enriched fractions (Figure 1e). Accordingly, higher amounts of RIP- and PARP-cleaved products were detected in HS samples (Figure 1e). Caspase-3, caspase-8 and caspase-9 activation was increased by more than twofold after hyperthemia (Figure 1f). Consistent with this increase, hyperthermia was also able to enhance Fas ligand-induced apoptosis, but failed to increase apoptosis induced by the PKC-inhibitor staurosporine (Supplementary Figure 3a). This result suggests that the mitochondrial pathway is likely dispensable to restore TRAIL-induced cell death during HS. To test this hypothesis, caspase-9 or BID were silenced. Although BID silencing attenuated TRAIL-induced apoptosis in MDA-MB-231 cells at 37 °C, it failed to inhibit apoptosis induced by TRAIL after a HS (Figure 1g). Loss of caspase-9 had no effect on these cells, irrespective of the temperature (Figure 1g). Consistent with a lack of requirement for mitochondrial activation, ectopic expression of Bcl-xL only slightly attenuated TRAIL-induced apoptosis during HS as compared with Mock-infected cells (Figure 1h). Moreover, restoration of TRAIL-induced apoptosis by HS was as efficient in the Bax-deficient prostate carcinoma cell line DU145 (Figure 1b), as in parental MDA-MB-231 cells pre-incubated with Bax channel blockers (Supplementary Figure 3b). By contrast and as expected, caspase-8 silencing completely abrogated apoptosis induced by TRAIL both at 37 °C and after HS (Figure 1i), indicating that reactivation of the mitochondrial pathway is dispensable for TRAIL signaling during HS.

### Hyperthermia impedes c-FLIP recruitment to the TRAIL DISC

**Figure 2:**
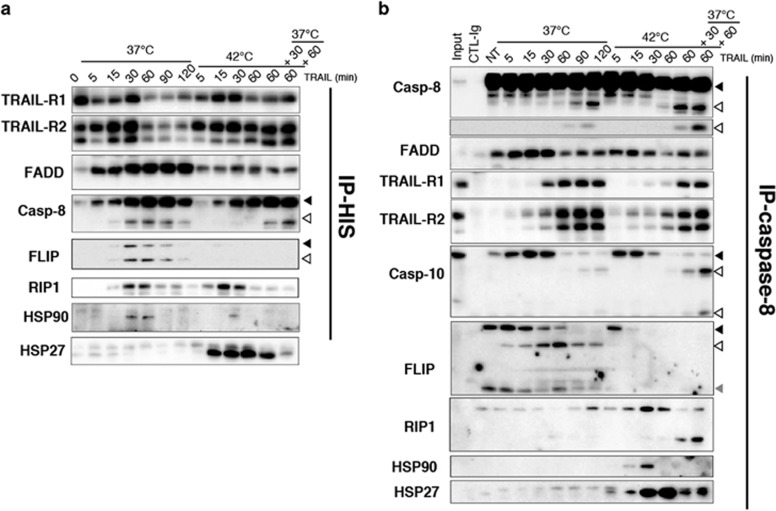
Hyperthermia prevents FLIP recruitment to the TRAIL DISC. (**a**) MDA-MB-231 cells, stimulated with 500 ng/ml His-TRAIL at 42 °C and/or at 37 °C for the indicated period of time, were lysed and the TRAIL DISC was immunoprecipitated using an anti-histidine antibody (see methods). DISC components associated with TRAIL were analyzed by immunoblotting as indicated. (**b**) MDA-MB-231 cells were stimulated as above and the initiator caspase-8 was immunoprecipitated. TRAIL DISC components were analyzed by immunoblotting. Alternatively, an isotype control antibody (CTL-Ig) was used for specificity. NT stands for non-treated cell extracts. (**a**, **b**) One representative blot is shown (*n*=3). Black arrows show uncleaved proteins, white arrows indicate cleaved proteins. Grey arrow shows the short c-FLIP isoform

As the mitochondrial pathway is not a prerequisite for HS and TRAIL synergistic effect, and because it has been proposed that HS may act at the level of plasma membrane,^26^ we decided to analyze TRAIL DISC composition during and after HS. As shown Figure 2a, TRAIL DISC formation and composition were significantly different in cells incubated at 37 °C as compared with cells incubated at 42 °C. Strikingly, although c-FLIP was co-recruited with caspase-8 within the TRAIL DISC at 37 °C, it was not recruited at 42 °C, even when cells were allowed to recover from the HS at 37 °C (Figure 2a). Consistently, a loss of c-FLIP/caspase-8 interaction was detected after caspase-8 immunoprecipitation in cells stimulated with TRAIL in HS condition (Figure 2b). In line with the gain of caspase activation and apoptosis induced by TRAIL after a HS, we observed an increase in caspase-8 and caspase-10 processing within the DISC, 120 min after TRAIL stimulation, and the cleavage of RIP1 (Figure 2).

HSPs inhibit apoptosis through their ability to interfere with mitochondria.^27^ However, a report suggested that HSP90 confers resistance to TRAIL through its ability to interact with c-FLIP and to increase c-FLIP recruitment within the DISC.^28^ HSP90 could be detected at 37 °C consistent with c-FLIP recruitment (Figure 2a). However, at 42 °C and therefore in the absence of c-FLIP, albeit to a lesser extent, HSP90 was still detected in the DISC (Figure 2a). Moreover, HSP90 was not observed at 37 °C in the caspase-8 pull-down despite the presence of c-FLIP, but was detected 5 and 15 min after TRAIL stimulation at 42 °C, a condition in which little c-FLIP remained associated with caspase-8 (Figure 2b). Contrary to HSP90, however, recruitment of HSP27 was consistently found in the TRAIL DISC at 42 °C but not at 37 °C (Figure 2b), and no HSP70 could be detected in the DISC, irrespective of the temperature (not shown).

To sort out whether the presence of HSP27 or HSP90 within the DISC might be relevant, these inducible HSPs were silenced. Although the silencing of HSP27 enhanced TRAIL both at 37 °C and 42 °C, silencing of HSP70, HSP90*α* or HSP90*β* had no significant effect, irrespective of the temperature (Supplementary Figure 3c and not shown). Silencing simultaneously both isoforms of HSP90, however, attenuated apoptosis induced by TRAIL during HS but not at 37 °C (Supplementary Figures 3c and d). HSP90 is a chaperone of RIP1, and is known to stabilize this kinase.^29^ To exclude the possibility that the effects of HSP90*α/β* silencing might require RIP1, the kinase was silenced. Unlike HSP90*α/β*, silencing of RIP1 was unable to alter TRAIL-induced cell death during HS (Supplementary Figure 3d), suggesting that HSP90's regulatory properties are independent of RIP1. Analysis of caspase activation by immunoblotting in MDA-MB-231 cells silenced for HSP27, HSP90*α/β* or RIP1 demonstrated that these proteins are unable to regulate early events of TRAIL-induced cell death during HS (Supplementary Figure 3e). Neither caspase-8 nor c-FLIP cleavage was altered in cells lacking HSPs or RIP1, irrespective of the temperature. However, as expected, in cells silenced for HSP27, activation of caspase-9 and caspase-3 was significantly increased upon TRAIL stimulation, both at 37 and 42 °C (Supplementary Figure 3e), indicating that HSP27 mainly inhibits TRAIL signaling at the mitochondrial level. Our results show that HSPs are not directly involved in regulating HS-induced TRAIL sensitization at the DISC level.

### Hyperthermia induced c-FLIP insolubilization occurs in an ubiquitination- and phosphorylation-independent manner and is not regulated by c-FLIP DED2 chain assembly domain

**Figure 3:**
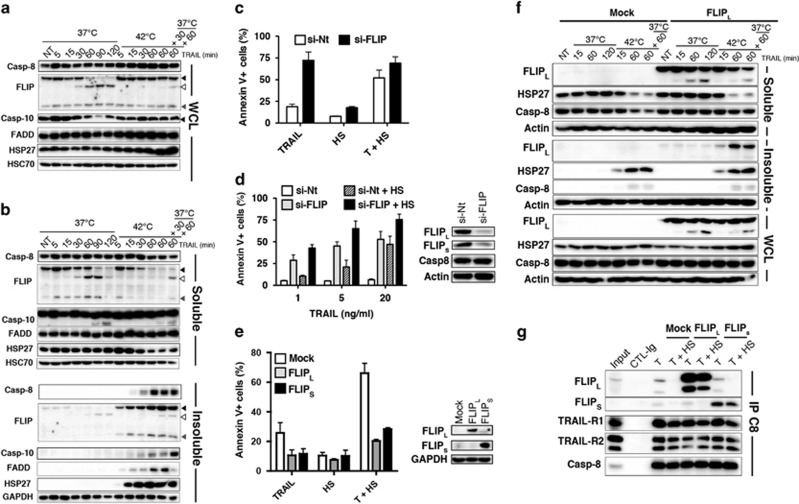
Hyperthermia-mediated cytosolic c-FLIP depletion is sufficient to sensitize tumor cells to TRAIL apoptosis. (**a**) MDA-MB-231 cells were treated with 500 ng/ml His-TRAIL or not (NT) for the indicated times and temperatures, and c-FLIP expression was analyzed from whole-cell lysate (WCL) by immunoblotting together with caspase-8, -10 and FADD. HSC70 and HSP27 were used as loading and heat shock controls, respectively. Black arrows show uncleaved proteins, white arrows indicate cleaved proteins. Grey arrow shows the short c-FLIP isoform. (**b**) MDA-MB-231 cells were stimulated as above and lysed in NP40 1% lysis buffer. After centrifugation, the supernatants (soluble) and the pellets resuspended in a buffer containing 1% SDS (insoluble), were loaded and indicated proteins were analyzed by immunoblotting. (**c**, **d**) MDA-MB-231 cells were transfected with non-targeting (si-Nt) or FLIP (si-FLIP) targeting siRNAs and stimulated 72 h after transfection with (**c**) 50 ng/ml or (**d**) increasing concentrations of His-TRAIL and apoptosis (Annexin V) was measured by flow cytometry. FLIP expression levels were controlled by immunoblotting. (**e**) MDA-MB-231 cells stably expressing c-FLIP_L_ or c-FLIP_S_ or infected with an empty vector (Mock) were stimulated with 50 ng/ml His-TRAIL as above and apoptosis (Annexin V) was measured by flow cytometry. FLIP expression levels were controlled by immunoblotting. (**f**) Stably expressing c-FLIP_L_ or Mock-infected MDA-MB-231 cells were stimulated or not (NT) as indicated with 500 ng/ml His-TRAIL for 15–120 min and expression of c-FLIP_L_ was analyzed by immunoblotting in whole-cell lysates (WCL), as well as cytosolic and insoluble NP40 detergent fractions, as described above. (**g**) MDA-MB-231 stably expressing c-FLIP_L_, c-FLIP_S_ or mock-infected (Mock) cells were stimulated with 500 ng/ml His-TRAIL for 30 min at 37 °C or at 42 °C (T+HS) and caspase-8 was immunoprecipitated for analysis of TRAIL DISC composition by immunoblotting. (**a**, **b**, **f**, **g**) One representative blot is shown (*n*=3)

Hyperthermia has been proposed to induce ubiquitination-dependent c-FLIP degradation through the proteasome, allowing restoration of TRAIL- and mapatumumab-induced cell death.^17^ In MDA-MB-231 stimulated with TRAIL during HS, c-FLIP isoforms remain highly expressed for up to 2 h as evidenced in whole-cell lysates (Figure 3a). We thus hypothesized that the impairment of c-FLIP recruitment to TRAIL DISC during HS (Figure 2) is unlikely due to its mere degradation by the proteasome. In line with our hypothesis, although c-FLIP levels dropped after TRAIL treatment during HS in the detergent-soluble fraction (Figure 3b), c-FLIP rapidly accumulated in the insoluble fraction (Figure 3b). Interestingly, caspase-8, caspase-10 and FADD were also found in the insoluble fraction. Yet, contrary to c-FLIP, a large proportion of caspase-10 and FADD, and to a lesser extent of caspase-8, remained in the soluble fraction 2 h after TRAIL stimulation (Figure 3b). Its high susceptibility to insolubility, most likely rendered c-FLIP unavailable for DISC recruitment and thus contributed to TRAIL-induced apoptosis restoration during HS. Accordingly, silencing of c-FLIP alone was sufficient to restore TRAIL sensitivity to similar extent as cells exposed to a HS (Figure 3c), even at low TRAIL concentrations (Figure 3d). On the other hand, increasing c-FLIP levels, irrespective of the isoform, inhibited TRAIL-induced cell death, both at 37 °C or after a HS (Figure 3e). The increase of c-FLIP expression in these cells was sufficient to maintain enough c-FLIP levels within the cytosol during the course of the HS (Figure 3f), and to inhibit TRAIL DISC formation and apoptosis (Figures 3g and e, and Supplementary Figure 4a).

To exclude the possibility that loss of c-FLIP in the detergent insoluble fraction during HS required ubiquitination-mediated c-FLIP degradation,^18, 30^ point mutants targeting lysine 167 and 195 or threonine 166 were generated, and stably expressed in MDA-MB-231 cells, to monitor c-FLIP solubility (Supplementary Figure 4b). Like WT c-FLIP, ubiquitinylable-deficient and phosphorylation c-FLIP mutants translocated to the detergent insoluble fraction upon HS, indicating that neither ubiquitination, nor regulation of ubiquitination through c-FLIP phosphorylation contributed to c-FLIP insolubility upon HS. Lysine 106 and serine 193 c-FLIP mutants were used here as negative controls for proteosomal mediated degradation.^18, 19^ Regardless of the point mutation, c-FLIP mutants were as efficient as WT c-FLIP in inhibiting TRAIL-induced cell death during HS (not shown), even after a 2 h incubation time at 42 °C (Supplementary Figure 4c). Similar to WT c-FLIP, most of these mutants were expressed at high levels in MDA-MB-231 cells and a large proportion remained in the cytosol 60 min after HS, explaining their ability to inhibit TRAIL-induced cell death. Consistent with the demonstration that c-FLIP ubiquitination-mediated degradation is not required for depletion of c-FLIP from the cytosol, pre-incubation of MDA-MB-231 cells in the presence of MG132 failed to restore c-FLIP expression in the soluble fraction, regardless of *de novo* protein synthesis (Supplementary Figure 4d). However, inhibition of the proteasome by MG132 led to the accumulation of c-FLIP in the insoluble fraction, demonstrating that c-FLIP degradation occurs after its depletion from the cytosol. Insolubilization of c-FLIP was also independent of c-FLIP DED2 chain assembly motif, as the two chain assembly motif mutants (F114G and F114G/L115G)^31, 32^ were as efficiently depleted from the cytosolic fraction as WT c-FLIP (Supplementary Figure 5a). At the contrary, depletion of FL114/115G c-FLIP mutant was even more pronounced than WT c-FLIP during HS. Interestingly, this mutant was less efficient than WT c-FLIP in inhibiting TRAIL-induced cell death upon HS (Supplementary Figure 5b), again indicating that depletion of c-FLIP from the cytosol, alone, is sufficient to restore TRAIL-induced cell death.

### Restoration of c-FLIP in the cytosol enables c-FLIP recruitment to TRAIL DISC and inhibits apoptosis induced by TRAIL during HS

**Figure 4:**
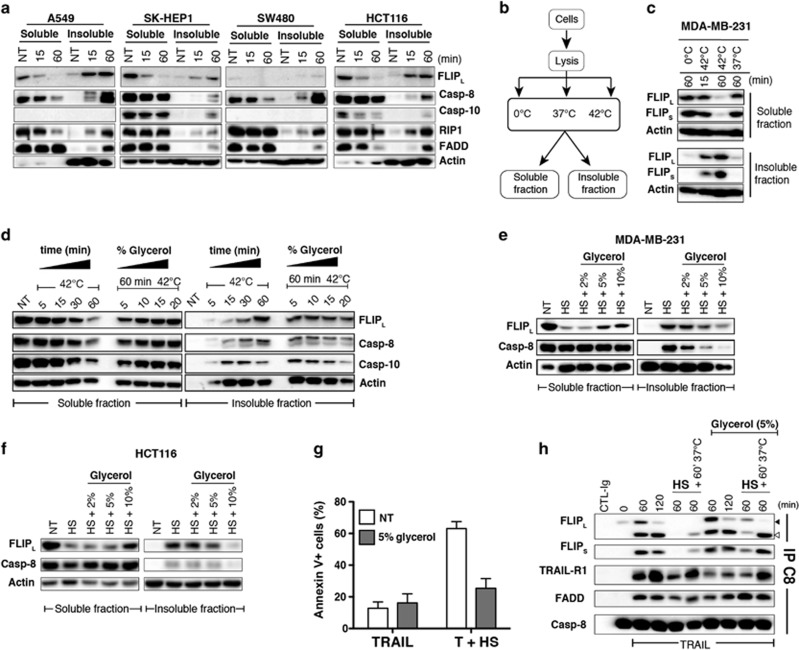
Hyperthermia-induced c-FLIP_L/S_ aggregation provokes c-FLIP depletion from the cytosol, thereby preventing its recruitment to the TRAIL DISC and enhancing apoptosis induced by TRAIL. (**a**) The lung carcinoma A549, the liver adenocarcinoma SK-HEP1, and the two colon carcinoma SW480 and HCT116 cell lines were incubated or not (NT) at 42 °C for 15 or 60 min and c-FLIP_L_ expression was analyzed in NP40 soluble and insoluble fractions by immunoblotting. (**b**) Schematic representation of the cell fractionation experiment performed in (**c**). MDA-MB-231 cells were lysed in NP40. Extracts were then either incubated on ice (0 °C) or at 37 °C for 60 min, or incubated for 15 or 60 min at 42 °C and soluble and insoluble fractions were separated by centrifugation to analyze FLIP expression levels by immunoblotting. (**d**) MDA-MB-231 cells were lysed in NP40 and incubated or not at 42 °C for the indicated times in the presence or absence of increasing amounts of glycerol (expressed here as % v/v). Expression levels of c-FLIP_L_, caspase-8 and caspase-10 were analyzed by immunoblotting. Actin was used as a loading control. (**e** and **f**) MDA-MB-231 or HCT116 cells were pre-incubated for 60 min with increasing amounts of glycerol and incubated or not at 42°C for 30 minutes. Cells were then lysed in NP40 and cell extracts and centrifuged to collect the soluble and the insoluble NP40 fractions. c-FLIP and caspase-8 content was analyzed by immunoblotting. (**g**) MDA-MB-231 cells were pre-incubated as above in the presence of 5% glycerol and stimulated or not 6 h in the presence of 50 ng/ml His-TRAIL at 37 °C or 42 °C (HS, see Figure 1a). Apoptosis (Annexin V) was measured by flow cytometry. (**h**) MDA-MB-231 cells were pre-incubated or not for 60 min with glycerol (5% v/v) and stimulated with 500ng/ml His-TRAIL at 37°C or at 42°C (HS). TRAIL DISC was analyzed 2 h after the onset of the stimulation by immunoblotting after immunoprecipitation of the caspase-8. Black arrows show uncleaved proteins, white arrows indicate cleaved proteins. (**a**, **c**, **d**, **e**, **f**, **h**) One representative blot is shown (*n*=3)

Translocation of c-FLIP during HS was also found in other tumor cell lines of various origin (Figure 4a). As observed in MDA-MB-231, disappearance of c-FLIP from the soluble fraction in these cells was always more efficient than depletion of FADD, RIPK1, caspase-8 or caspase-10, suggesting that enough DISC component remains in the cytosol to allow efficient TRAIL-induced cell death in the absence of c-FLIP. In order to determine whether the mere increase in temperature, but not sequestration of c-FLIP into subcellular compartments induced or not through HS-mediated MAPK signalling, is sufficient to induce c-FLIP aggregation and loss from the cytosol, cell lysates obtained from unstimulated MDA-MB-231 cells were incubated at 0, 37 or 42 °C for the indicated period of time, then centrifuged to separate NP40 soluble and insoluble fractions (Figure 4b) and samples were analyzed by immunoblotting. In cell extracts incubated at 42 °C, c-FLIP content decreased in the soluble fraction in a time-dependent manner to accumulate in the insoluble fraction (Figure 4c). As expected, incubation of cell lysates at 0 or 37 °C for 60 min failed to induce c-FLIP depletion from the cytosolic fraction. Insolubilization of c-FLIP, but also of initiator caspases, was most likely triggered through protein aggregation as addition of increasing amounts of glycerol before incubation at 42 °C reduced, in a dose-dependent manner, the amount of c-FLIP and caspase-8 present in the insoluble fraction and restored significant c-FLIP protein content in the cytosolic fraction (Figure 4d). Addition of glycerol on intact MDA-MB-231 or HCT116 cells prior to incubation at 42 °C also inhibited HS-induced c-FLIP aggregation as evidenced by the increase of c-FLIP_L_ in the cytosolic fraction (Figures 4e and f). As glycerol is able to prevent the loss of c-FLIP from the cytosol, we speculated that it might protect, at least partially, tumor cells from TRAIL-induced cell death after a HS. To address this question, MDA-MB-231 cells were incubated for 60 min in the presence of increasing amounts of glycerol before stimulation with TRAIL at 42 °C. Analysis of apoptosis induced in these conditions indicates that addition of glycerol prior stimulation protected cells from TRAIL-induced apoptosis during HS (Figure 4g) and restored c-FLIP recruitment to the TRAIL DISC (Figure 4h). Our results thus provide evidence that restoration of death receptor-induced cell death by hyperthermia is essentially mediated through c-FLIP aggregation.

## Discussion

Hyperthermia was first demonstrated to restore cell death induced by TNF*α* in the late 80s.^33, 34^ Only recently has hyperthermia been successfully exploited in the clinic with TNF*α* to treat limb soft tissue sarcomas with high response rates,^35^ or locally advanced cancers.^36^ Besides TNF*α*, hyperthermia can also promote Fas ligand and TRAIL-induced apoptosis.^25, 37, 38^ Accordingly, we show here that hyperthermia restores TRAIL pro-apoptotic signaling in a large panel of tumor cell lines. Yet, contrary to previous demonstrations pointing to a contribution of mitochondria,^37, 39, 40, 41^ our findings clearly demonstrate that the mitochondrial pathway is not a prerequisite because neither caspase-9-, Bid- or bax-deficiency nor Bcl-xL overexpression compromised TRAIL-induced cell death during HS. Lack of apparent contribution of caspase-9, in our settings, as opposed to Bid at 37 °C is most likely due to differential contribution of other mitochondrial pro-apoptogenic factors such as Smac/Diablo, whose release in the cytosol was shown to play an important contribution for TRAIL-induced cell death in MDA-MB-231 cells.^42^ Likewise, XIAP but not caspase-9 inhibition has recently been demonstrated to play a role in regulating TRAIL-induced apoptosis in HCT116 cells, described as type II cells.^43^

Irrespective of mitochondrial pro-apoptogenic factors, our results demonstrate that inhibition of c-FLIP recruitment within the TRAIL DISC is the main mechanism through which hyperthermia restores TRAIL-induced cell death. Accordingly, the loss of c-FLIP recruitment within the DISC was always associated with increased activation of initiator caspases. Overexpression of c-FLIP alone or inhibition of c-FLIP aggregation, using glycerol, was sufficient to restore c-FLIP recruitment within the DISC and to compromise TRAIL-induced apoptosis during HS. Moreover, c-FLIP silencing, alone, was sufficient to phenocopy the effects of HS, and combining c-FLIP silencing and HS induced no additional gain of function.

c-FLIP is probably the most important regulator of apoptosis induced by death receptors.^6^ This caspase-8 inhibitor is highly regulated in normal and tumor cells,^44, 45^ and highly susceptible to a plethora of compounds, rendering c-FLIP an interesting target for cancer therapy.^7, 46^ Hyperthermia has recently been proposed to enhance TRAIL- and mapatumumab-induced cell death through FLIP degradation.^17^ Although our results are in full agreement with the finding that c-FLIP is the main regulator targeted by hyperthermia allowing restoration of TRAIL sensitivity in resistant tumor cells, our data suggest that loss of c-FLIP within the TRAIL DISC is, however, not a direct consequence of its degradation, but rather of its aggregation and thereby its disappearance from the cytosol. Loss of c-FLIP from the cytosol was evidenced almost as early as 5 min after HS, much earlier than the onset of c-FLIP cleavage or c-FLIP degradation that was almost not observed in our settings. Depletion of c-FLIP from the cytosol also coincided with the loss of c-FLIP binding to caspase-8 after TRAIL DISC formation. Contrary to c-FLIP, however, caspase-8 was still recruited at 42 °C, consistent with the gain of pro-apoptotic function afforded by short incubation of the cancer cells at 42 °C in the presence of TRAIL.

Moreover, despite the fact that the ubiquitin-proteasomal pathway emerges as an important regulator of c-FLIP expression in tumor cells,^47^ our results demonstrate that neither phosphorylation or ubiquitination of c-FLIP nor inhibition of the proteasome inhibited c-FLIP depletion from the cytosol during hyperthermia, indicating that ubiquitination-mediated proteosomal degradation of c-FLIP is not required for its depletion from the cytosol.

Our results show that the depletion of c-FLIP from the cytosol and its recovery in an insoluble cellular fraction after a heat shock is most likely triggered by its aggregation. Hyperthermia is known to induce protein aggregation, leading eventually to cell death.^48, 49^ In agreement with these findings, not only the disappearance of c-FLIP but also, to a lesser extent, of initiator caspases including caspase-8 and caspase-10 from the non-ionic detergent soluble fraction after hyperthermia was detected in all the tumor cell lines studied. However, contrary to the caspase-8 or the caspase-10, insolubilization of c-FLIP led to full disappearance of the protein from the cytosol suggesting that c-FLIP may be more thermolabile than caspase-8 or caspase-10, or that complete depletion induced by HS is due to lower expression levels of c-FLIP, as compared to caspase-8 or FADD.^32^ Preferential depletion of c-FLIP was not due to its DED2 chain assembly motif, two amino acids found to be essential for FADD binding and recruitment to the TRAIL DISC.^31, 32^

Keeping in mind that the amounts of c-FLIPs are most of the time much lower than caspase-8 ^32^ or that c-FLIP recruitment within the TRAIL DISC is 5 to 10 times lower than caspase-8,^31, 50^ and that at equivalent concentration, caspase-8, like FADD, as shown by the use of recombinant GST-fused proteins (Supplementary Figure 6a), are found as efficiently as c-FLIP in the insoluble fraction of the bacterial cell extracts after a heat shock, preferential loss of c-FLIP from the cytosolic fraction is likely to be due, at least in part, to its low intracellular steady state level. Yet, it can't be excluded that in addition, its higher thermolability, as predicted from its amino acid sequence^51^ (Supplementary Figure 6b). might also play a role.

Whatsoever, the addition of glycerol, a compound shown to inhibit protein aggregation,^49^ before hyperthermia prevented, not only the loss of capase-8 and FADD from the cytosolic fraction but also the loss of c-FLIP, enabling its recruitment within the DISC and conferring partial protection to TRAIL-induced cell death.

Altogether our results provide the first demonstration that aggregation of c-FLIP induced by hyperthermia, but not degradation, impairs c-FLIP recruitment to TRAIL DISC and thus enhances or restores TRAIL-induced cell death in resistant cells through depletion of cytosolic c-FLIP reservoir. Keeping in mind that extensive research is being pursued worldwide to use TRAIL or TRAIL derivatives in the clinic and that c-FLIP isoforms are often highly expressed in tumor cells,^7^ inhibiting c-FLIP solubility with locally applied or whole-body hyperthermia could be relevant to cancer TRAIL-based therapies.^2, 17^

## Materials and Methods

### Ligand production and chemicals

His-tagged TRAIL and FasL were produced and used as described previously.^52^ Staurosporine, glycerol, nonidet P-40 (NP40) and sodium dodecyl sulfate (SDS) were purchased from Sigma-Aldrich (Lyon, France). Bax channel blocker was from Santa Cruz Biotechnology (Tebu-bio, Le Perray en Yvelines, France). MG-132 (Cat# 1748) was from Tocis (Bristol, United Kingdom). The pan-caspase inhibitor z-VAD-fmk and cycloheximide (Cat# ALX-380-269) were from Enzo Life Science (Villeurbanne, France).

### Antibodies

For western blot analysis, anti-TRAIL-R1 (Cat# AB16955) and TRAIL-R2 (Cat# AB16942) were purchased from Chemicon (Millipore, Molsheim, France). Anti-FADD (Cat# 610400) and RIP1 antibodies (Cat# 551041) were obtained from Transduction Laboratories (BD Biosciences, Le Pont de Claix, France). Anti-caspase-8 (clone 5F7) and caspase-10 (clone 4C1) antibodies were obtained from Medical & Biological Laboratories (Clinisciences, Montrouge, France). Antibodies against BID (Cat# 2002), caspase-3 (clone 8G10), cleaved caspase-9 (clone Asp315), Histone H3 (clone D1H2), p-HSP27 S82 (clone D1H2), cleaved PARP (clone D65E10) and Vimentin (clone D21H3) were obtained from Cell Signaling (Millipore). Anti-caspase-8 (clone C20), GAPDH (clone 0411), HSC70 (clone B-6), TNFR1 (clone H5), and Ubiquitin (clone P4D1) antibodies were from Santa Cruz Biotechnology. Anti-FLIP (clone 7F10), HSP27 (clone G3.1), Anti-p-HSP27 S15 (Cat# ADI-SPA-525), p-HSP27 S78 (Cat# ADI-SPA-523), HSP70 (clone C92F3A-5) and HSP90*β* (clone K3705) antibodies were from Enzo Life Science. Anti-FLIP antibody (clone Dave-2) was from Adipogen (Coger, Paris, France). Anti-Bcl-xL (clone E18) and anti-HSP90*α* (clone D7a) antibodies were purchased from ABCAM (ABCAM, Paris, France). The anti-Histidine (clone AD1.1.10) was from AbD serotec (Bio-Rad, Marnes-la-Coquette, France). HRP-conjugated anti-rabbit or mouse antibodies were from Jackson ImmunoResearch (Interchim, Montluçon, France). HRP-conjugated anti-mouse IgG1-, Ig2a- and Ig2b-specific antibodies were from Southern Biotech (Clinisciences). For flow cytometry experiments, the anti-TRAIL-R1 (clone wB-K32), and TRAIL-R2 (clone B-L27), TRAIL-R3 (clone wB-B44) and TRAIL-R4 (clone wB-P30) antibodies were kindly provided by Diaclone (Besançon, France). The secondary antibody was an Alexa-488 coupled-goat anti-mouse from Molecular Probes (Life technologies, Saint Aubin, France).

### Cell culture

MDA-MB-231 (human mammary adenocarcinoma), A549 (human lung adenocarcinoma), SK-HEP-1 (human hepatocellular carcinoma), DU145 (human prostate adenocarcinoma) and the two human colon adenocarcinomas HCT116 and SW480 cell lines were cultured with high-glucose Dulbecco's modified Eagle's medium provided by Dutscher (Dutscher, Brumath, France) supplemented with 10% fetal bovine serum (Dutscher). PANC-1 (human pancreatic carcinoma) cell line was cultured in RPMI 1640 (Roswell Park Memorial Institute medium) provided by Dutscher and supplemented with 10% fetal bovine serum. All these cell lines were grown in an incubator at 37 °C and 5% CO_2_.

### Retrovirus production and cell transduction

The retroviral vector pBABE-puro encoding Bcl-xL was kindly provided by Dr Jerry Chipuk (Icahn Medical Institute, New York, NY, USA), WT VSV tagged c-FLIP_L_ and c-FLIP_S_ have previously been described.^53^ Mutated VSV-FLIP_L_ were produced by site-directed mutagenesis using pBABE-puro-WT-VSV-FLIP_L_ and the following primers: K106R forward 5′-GAG-ATT-GGT-GAG-GAT-TTG-GAT-AGA-TCT-GAT-GTG-TCC-TCA-TTA-AT-3′ reverse 5′-ATT-AAT-GAG-GAC-ACA-TCA-GAT-CTA-TCC-AAA-TCC-TCA-CCA-ATC-TC-3′ T166A forward 5′-CAC-AGA-ATA-GAC-CTG-AAG-GCA-AAA-ATC-CAG-AAG-TAC-AAG-3′ reverse 5′-CTT-GTA-CTT-CTG-GAT-TTT-TGC-CTT-CAG-GTC-TAT-TCT-GTG-3′ T166D forward 5′-CAC-AGA-ATA-GAC-CTG-AAG-GAT-AAA-ATC-CAG-AAG-TAC-AAG-C-3′ reverse 5′-GCT-TGT-ACT-TCT-GGA-TTT-TAT-CCT-TCA-GGT-CTA-TTC-TGT-G-3′ K167R forward 5′-CAG-AAT-AGA-CCT-GAA-GAC-ACG-AAT-CCA-GAA-GTA-CAA-GCA-G-3′ reverse 5′-CTG-CTT-GTA-CTT-CTG-GAT-TCG-TGT-CTT-CAG-GTC-TAT-TCT-G-3′ T166A/K167R forward 5′-CCA-CAG-AAT-AGA-CCT-GAA-GGC-ACG-AAT-CCA-GAA-GTA-CAA-GCA-G-3′ reverse 5′-CTG-CTT-GTA-CTT-CTG-GAT-TCG-TGC-CTT-CAG-GTC-TAT-TCT-GTG-G-3′ T166D/K167R forward 5′-CAC-AGA-ATA-GAC-CTG-AAG-GAT-CGA-ATC-CAG-AAG-TAC-AAG-CAG-3′ reverse 5′-CTG-CTT-GTA-CTT-CTG-GAT-TCG-ATC-CTT-CAG-GTC-TAT-TCT-GTG-3′ S193A forward 5′-GCA-GCA-ATC-CAA-AAG-GCT-CTC-AAG-GAT-CCT-TCA-AAT-AAC-3′ reverse 5′-GTT-ATT-TGA-AGG-ATC-CTT-GAG-AGC-CTT-TTG-GAT-TGC-TGC-3′ S193D forward 5′-GCA-GCA-ATC-CAA-AAG-GAT-CTC-AAG-GAT-CCT-TCA-AAT-AAC-3′ reverse 5′-GTT-ATT-TGA-AGG-ATC-CTT-GAG-ATC-CTT-TTG-GAT-TGC-TGC-3′ K195R forward 5′-CAA-GCA-GCA-ATC-CAA-AAG-AGT-CTC-AGG-GAT-CCT-TCA-AAT-3′ reverse 5′-ATT-TGA-AGG-ATC-CCT-GAG-ACT-CTT-TTG-GAT-TGC-TGC-TTG-3′ F114G forward 5′-TCT-GAT-GTG-TCC-TCA-TTA-ATT-GGC-CTC-ATG-AAG-GAT-TAC-ATG-GGC-3′ reverse 5′-GCC-CAT-GTA-ATC-CTT-CAT-GAG-GCC-AAT-TAA-TGA-GGA-CAC-ATC-AGA-3′ F114G/L115G forward 5′-TCT-GAT-GTG-TCC-TCA-TTA-ATT-GGC-GGC-ATG-AAG-GAT-TAC-ATG-GGC-3′ reverse 5′-GCC-CAT-GTA-ATC-CTT-CAT-GGG-CGG-AAT-TAA-TGA-GGA-CAC-ATC-AGA-3′. All constructs were checked by sequencing and used to generate stably expressing FLIP_L_ mutants as follows. Cells were transduced for 16 h with viral supernatants containing polybrene (8 mg/ml), washed in PBS and cultured in complete medium containing puromycin (2.5 *μ*g/ml) obtained from Invivogen (Invivogen, Toulouse, France).

### Treatments with hyperthermia and TRAIL

Cells were treated with the indicated concentration of His-TRAIL in supplemented DMEM and heated at 42 °C for 1 h or the indicated time in a preheated water bath or at 37 °C in an incubator. Cells were then incubated for 5 h or the indicated time with 5% CO_2_ at 37 °C before analysis.

### Hoechst analysis

Apoptosis was assessed by Hoechst staining (20 *μ*g/ml) and determination of the percentage of condensed and/or fragmented nuclei from at least 300 cells per conditions on three different fields. Experiments were repeated at least three times.

### Annexin V analysis

Annexin V-FITC staining kit was purschased from Miltenyi Biotec (Miltenyi Biotec, Paris, France) and used according to the manufacturer's instructions. Stained cells were analyzed with a BD LSR2 flow cytometer (BD Biosciences). The percentage of Annexin V-positive cells was calculated as the number of cells demonstrating Annexin V staining (PI negative or positive) divided by the total number of cells examined. Experiments were repeated at least three times.

### Caspases activity analysis

Cells (10^3^) were implanted in 96-well plates. Twenty-four hours after, cells were stimulated with 50 ng/ml His-TRAIL at 37 °C for the indicated time. Alternatively, cells were stimulated with TRAIL at 42 °C for 1 h then were incubated or not at 37 °C for the remaining time (1–7 h), before caspase activity analysis. Caspases-3/-7 (DEVDase, Cat# G8090), Caspase-8 (IETDase, Cat# G8200) and Caspase-9 (LHDase, Cat# G8210) activities were measured by luminometry with a commercial kit obtained from Promega (Promega France, Charbonnière, France) according to the manufacturer's protocol. Experiments were repeated at least three times.

### Gene silencing by siRNA

siRNA have been transfected with Interferin purshased from Polyplus (Polyplus transfection, Illkirch, France), according to the manufacturer's protocol. siRNA directed against BID (Cat# L-004387-00), Caspase-9 (Cat# L-003309-00), Caspase-8 (Cat# L-003466-00), HSP90A (Cat# L-005186-00), HSP90B (Cat# L-005187-00), RIP1 (Cat# L-004445-00), FLIP (Cat# L-003772-00) and non-targeting (Cat# L-001810-10-05) were obtained from Dharmacon (Enzo Life Science). siRNA directed against HSP27 (Cat# AM16708) was purchased from Ambion (Life Technologies). siRNA directed against HSP70 (Cat# SASI_Hs01_00051449) was purchased from Sigma-Aldrich.

### Immunoprecipitation

For DISC analysis, 50 × 10^6^ MDA-MB-231 cells per T175 cm^2^ flasks were stimulated with 12 ml of supplemented DMEM containing 500 ng/ml His-TRAIL for the indicated time and temperature. Cells were then washed with cold PBS, snap frozen and lysed with 1 ml lysis buffer containing 1% NP40, 20 mM Tris-HCl pH 7.5, 150 mM NaCl and 10% glycerol. Lysates were pre-cleared with Sepharose 6B (Sigma-Aldrich) for 1 h at 4 °C with on a rotating wheel and then immunoprecipitated overnight with G-protein Sepharose beads (Cat# 17-0618-01, GE Healthcare, Dutscher) in the presence of 2 *μ*g of anti-Histidine, Caspase-8 (clone C20) or corresponding Ig isotype control (CTL-Ig). Beads were then washed three times with 1 × lysis buffer, and immunoprecipitates were eluted with SDS gel loading buffer (Tris-HCl 63 mM, SDS 1%, phenol red 0.03%, glycerol 10% and DTT 100 mM of pH6.8), boiled for 5 min and processed for immunoblotting.

### Lysates and fractionation

Cells were treated as indicated and washed in cold PBS. Whole-cell lysis was performed using the SDS gel loading buffer. Samples were sonicated and boiled for 5 min before loading and immunoblotting analysis. For soluble/insoluble fractionation experiments, cells were lysed for 20 min on ice in a non-ionic detergent containing buffer composed of 1% NP40, 20 mM Tris-HCl pH 7.5, 150 mM NaCl and 10% glycerol. Lysates were then centrifuged 12 min at 10 000 r.c.f. and supernatants (soluble fraction) or the pellet (insoluble fraction) were recovered in 1 × final concentration of SDS gel loading buffer and processed as above for immunoblot analysis. Alternatively, for glycerol studies, NP40 containing lysis buffer was used without or with increasing concentrations of glycerol as indicated.

### Western blotting

Immunoprecipitates or cell lysates obtained from WCL or cell fractionation were resolved by SDS-PAGE and transferred to PVDF membranes (Amersham, Biosciences, Les Ullis, France). Nonspecific binding sites were blocked by incubating membranes for 1 h in PBS containing 0.01% of Tween 20 and 5% (w/v) dried skimmed milk (PBS-Tm). Immunoblots were then incubated with specific primary antibody diluted in PBS-Tm for 2 h at room temperature or overnight at 4 °C, washed three times in PBS-T for 10 min. Membranes were then incubated with corresponding HRP-conjugated secondary antibody in PBS-T for 1 h and washed three times followed by a chemiluminescence detection with the Western Bright Quantum kit (Advansta, Menlo Park, CA, USA). Protein expression levels were detected with ChemiDoc MP gel imager (Bio-Rad) or using X-ray films.

## Abbreviations

BID: BH3 interacting-domain death agonist
CHX: ycloheximide
DD: death domain
DED: death effector domain
DISC: death-inducing signalling complex
DR: death receptor
FADD: Fas-associated death domain
FLIP: Fas-associated death domain-like interleukin-1*β*-converting enzyme-like inhibitory protein
HS: heat shock
HSP: heat shock protein
NF-κB: nuclear factor-κB
PARP: poly-(ADP-ribose) polymerase
RIP: eceptor interacting protein
ROS: reactive oxygen species
SD: standard deviation
SDS: sodium dodecyl sulphate
siRNA: short interfering RNA
si-Nt: non-targeting siRNA molecule
TNF: tumour necrosis factor
TRAIL: tumour necrosis factor-related apoptosis-inducing ligand
z-VAD-fmk: carboxybenzyl-VAD-fluoromethyl ketone
