# Supplementary material for: Hyperthermia restores apoptosis induced by death receptors through aggregation-induced c-FLIP cytosolic depletion
Source: Cell Death Dis. 2015 Feb 12;6(2):e1633–. doi: 10.1038/cddis.2015.12 (PMC4669817; doi:10.1038/cddis.2015.12)
Supplement: Supplementary Information [file cddis201512x1.doc]

Supplementary Methods :

**Cell culture**

CaSki (human cervix carcinoma), HeLa (human cervix adenocarcinoma), HT-29 (human colon adenocarcinoma), MDA-MB-468 (human mammary adenocarcinoma), MIA PaCa-2 (human pacreatic carcinoma), H1703 (human lung carcinoma) and SiHa (human cervix carcinoma) cell lines were cultured with high-glucose DMEM (Dulbecco’s modiﬁed Eagle’s medium medium) provided by Dutscher (Dutscher, Brumath, France) supplemented with 10% fetal bovine serum (Dutscher). AGS (human stomach adenocarcinoma), RL (non-Hodgkin's lymphoma) cell lines were cultured RPMI 1640 (Roswell Park Memorial Institute medium) provided by Dutscher and supplemented with 10% fetal bovine serum. All these cell lines were grown in an incubator at 37°C and 5% CO2.

**Viability experiment**

5 x 10^4^ cells per well were seeded in a 96-wells plate and stimulated with increasing concentrations of His-TRAIL (from 0 to 25 000 ng/ml) for 24 h. Cell viability was determined by methylene blue staining as follows. Non-adherent cells were washed twice in PBS. Remaining adherent cells were then fixed for 20 minutes in a PBS solution containing 2% paraformaldehyde, stained for 20 min using a borate buffer staining solution (H_3_BO_3_ 100 mM; Na_2_BO_7_ 25 mM and NaCl 120 mM) containing 0,5% methylene blue. Experiments were repeated at least three times.

**GST tagged protein solubility**

pGEX-6-Caspase-8 (ref : DU30022), pGEX-6-FADD (ref : DU30001) and pGEX-6-c-FLIP_L_ (ref : DU22247) were purchased from the PPU (Protein Phosphorylation and Ubiquitylation Unit) of the university of Dundee (Scotland). These GST-expressing vectors were transformed into the *Escherichia coli* strain BL21 and GST-tagged-c-FLIP_L_, GST-pro-caspase-8 or GST-FADD were produced using 0.5 mM IPTG O/N. After induction, cell pellets were resuspended in an hypotonic buffer (50 mM sodium phosphate, 300 mM NaCl, 10 mM Imidazole, 5 mM DTT, lyzozyme 1 mg/ml, pH 7.5), and bacterial cells were lyzed after 5 freeze and thaw cycles followed by a 2 min sonication time at amplitude 10%. Proteins were further purified by centrifugation for 30 min 40 000 rcf after addition of 30 % of precondensed TX-114 (12%). Proteins samples were then subjected to a heat shocked for 15 or 60 minutes, or left 60 minutes at 37°C, then centrifugated 12 minutes for 10 000 rcf before loading the supernatants (Soluble) or the pellet (Insoluble) on a SDS-PAGE and immunoblotting.

Supplementary figures :

**Figure S1. Effects of temperature variation on TRAIL-induced apoptosis and induction of heat shock proteins.**

(a) MDA-MB-231 cells were stimulated with 100 ng/ml of His-TRAIL for 1 h at 37°C, 39°C or 42°C and then incubated for 5 h at 37°C. Apoptosis was measured by Hoechst staining. (b) Cells were stimulated in hyperthermic (HS) or in cold conditions at 0°C on ice (CS) as above and apoptosis was quantified by Hoechst. (a, b) SD error bars from at least 3 independent experiments are shown. (c) MDA-MB-231 cells were incubated or not at 42°C for 30 min, 1 h and allowed or not to recover at 37°C for the indicated period of time. Expression levels of inducible HSPs was monitored by immunoblot. (d) MDA-MB-231 cells, were incubated at 42°C for the indicated period of time and allowed or not to recover for 30 minutes at 37°C, then lysed using a non-ionic detergent, NP40. After centrifugation, the cytosolic fraction (Soluble) and the insoluble fraction (Insoluble) were analyzed by immunoblot as indicated. Immunoblotting of caspase-3, RIP1 and Histone H3 were used as controls for soluble and insoluble fraction quality. HSC70 was used as a loading control. (c, d) One representative blot is shown (n=3).

**Figure S2**. **Sensitivity to hyperthermia-mediated TRAIL-induced apoptosis restoration correlates with high FLIP_L_ expression levels.**

(a) TRAIL receptor expression levels (grey line), compared to control isotype staining (filled curve), were analysed by immunostaining and flow cytometry in the indicated cells lines. Cell lines in which hyperthermia restored or enhanced TRAIL-induced apoptosis are labelled in red, non-responding cells are shown in blue. (b) Protein expression levels of TRAIL DISC components, caspases and HSPs in the indicated cancer cell lines were analyzed by immunoblot. One representative blot is shown (n=3). (c) Hyperthermia-responding and non-responding cell lines were stimulated for 16 h at 37°C with increasing amounts of His-TRAIL and cell viability was measured by methylene blue staining. (d) Indicated hyperthermia non-responsive cancer cell lines were stimulated or not with 100 ng/ml (H1703, MIA PaCa-2, HCT116) or 500 ng/ml (AGS) His-TRAIL at 37°C (TRAIL, white bars), in hyperthermic conditions (T + HS, black bars) or without TRAIL (HS, grey bars) as described (Fig. 1a), and apoptosis was measured by Hoechst staining. (c, d) SD, error bars from at least 3 independent experiments are shown.

**Figure S3**. **Hyperthermia-induced sensitization to** **TRAIL is independent of mitochondria and HSPs.**

(a) MDA-MB-231 cells were stimulated with His-TRAIL (100 ng/ml), FasL (100 ng/ml) or staurosporine (STS, 1 μg/ml) for 16 h at 37°C (white bars) or after a HS (1 h at 42°C, followed by an incubation at 37°C for 15 h, black bars). Apoptosis was measured by Hoechst staining. (b) MDA-MB-231 cells were treated as described in Fig. 1a with 100 ng/ml of His-TRAIL in the presence or absence of 5 μM Bax channel blockers (BCB). Apoptosis was measured as above. (c) MDA-MB-231 were transfected with non-targeting (si-Nt) or HSP27 (si-HSP27), HSP70 (si-HSP70) or HSP90α and/or β (si-HSP90) targeting siRNAs and stimulated 72 h after transfection with 50 ng/ml of His-TRAIL. Apoptosis was measured by Hoechst staining. Right panels, immunoblots of HSPs expression levels. (d) MDA-MB-231 cells were transfected with non-targeting (si-Nt) or HSP90α and HSP90β (si-HSP90α/β) or RIP1 (si-RIP1) targeting siRNAs and apoptosis induced by HS, TRAIL or TRAIL and HS was measured by hoechst. Corresponding HSP90 and RIP expression levels are shown on the right. (e) MDA-MB-231 cells were transfected and stimulated as described above, and caspase-3, -8, -9 and c-FLIP processing and/or cleavage was monitored by immunoblot. Expression levels of RIP1, HSP90 and HSP27 are shown as controls of siRNA efficiency. Black arrows indicate the proforms of caspase-8, caspase-9 and the uncleaved form of c-FLIP_L_. White arrows show p43 c-FLIP_L_ fragment and caspase-9 cleaved products. Grey arrow shows the short c-FLIP isoform. One representative blot is shown (n=3). (a, b, c, d) Error bars represent SD from at least 3 independent experiments (*, P <0.05; ***, P<0.001).

**Figure S4**. **Depletion of c-FLIP from the cytosol during hyperthermia is not triggered by its phosphorylation, ubiquitination or degradation.**

(a) MDA-MB-231 cells, stably infected with a retroviral vector encoding FLIP_L_ or an empty vector (Mock), were stimulated with 500 ng/ml His-TRAIL for the indicated times and temperatures. TRAIL DISC content was then analyzed by immunoblotting after immunoprecipitation of the caspase-8. Black arrows show uncleaved proteins, white arrows indicate cleaved proteins. (b) MDA-MB-231 cells expressing stably WT, phosphorylation and/or ubiquitination FLIP_L_-mutants were incubated for 30 min at 37°C (NT) or 42°C (HS). Then, proteins obtained from whole cell lysates (WCL), or NP40 non ionic detergent’s soluble fraction (Soluble) and corresponding remaining insoluble fraction (insoluble), were resolved on SDS-page and analyzed by immunoblot for c-FLIP_L_ content. Actin was used as a loading control. (c) Above mentioned MDA-MB-231 FLIP_L_ and FLIP_L_-mutant expressing cells were treated with 500 ng/ml His-TRAIL for 2 h at 42°C and further incubated for 4 h at 37°C before analysis of apoptosis using annexin V staining and flow cytometry. Error bars represent SD from at least 3 independent experiments. (d) Parental MDA-MB-231 cells were pre-treated or not with 10 μM/ml proteasome inhibitor MG-132 and/or 10 mg/ml protein synthesis inhibitor cycloheximide (CHX) for 1h. Cells were incubated for the indicated times at 42°C, lysed in NP40 to collect the soluble (Soluble) and the insoluble (Insoluble) fractions. c-FLIP_L_ and c-FLIP_S_ expression levels were analyzed by immunoblot. Actin served as a loading control. (a, b, d) One representative blot is shown (n=3).

**Figure S5. c-FLIP DED2 chain assembly domain is not required for cytosolic c-FLIP depletion upon hyperthermia.**

(a) MDA-MB-231 cells expressing stably WT, F114G or F114G/L115G DED2 chain assembly domain c-FLIP_L_ mutants or an empty retroviral vector (Mock) were incubated for the indicated times at 42°C and c-FLIP_L_ content in the soluble (cytosolic) or in the insoluble fraction was analyzed by immunoblot. One representative blot is shown (n=3). (b) Indicated cells were stimulated with 50 ng/ml His-TRAIL at 37°C or 42°C (HS) and annexin V staining was monitored 6 h after the onset of the stimulation by flow cytometry. SD, error bars from at least 3 independent experiments.

**Figure S6. Thermolability of c-FLIP, DISC components and precipitation of the corresponding GST-fused recombinant proteins after HS.**

(a) Thermal Index inferred from the amino acid composition of the indicated proteins using the Tm Index program available at http://tm.life.nthu.edu.tw/ (Ku et al. Comput Biol Chem. 2009 Dec;33 (6):445-50). (b) Recombinant GST-c-FLIP, GST-FADD and GST-Casp-8 (capase-8) cleared bacterial cell extracts obtained after 5 cycles of freeze and thaw in an hypotonic buffer, were left 60 minutes at 37°C or incubated 15 and 60 minutes at 42°C, as indicated. Cell extracts were then centrifugated and the supernatant (Soluble) was recovered for immunoblot analysis. The pellet (insoluble) was resuspended in loading buffer before analysis as above.
