# Supplementary figures and images for: Hyperthermia restores apoptosis induced by death receptors through aggregation-induced c-FLIP cytosolic depletion
Source: Cell Death Dis. 2015 Feb 12;6(2):e1633–. doi: 10.1038/cddis.2015.12 (PMC4669817; doi:10.1038/cddis.2015.12)

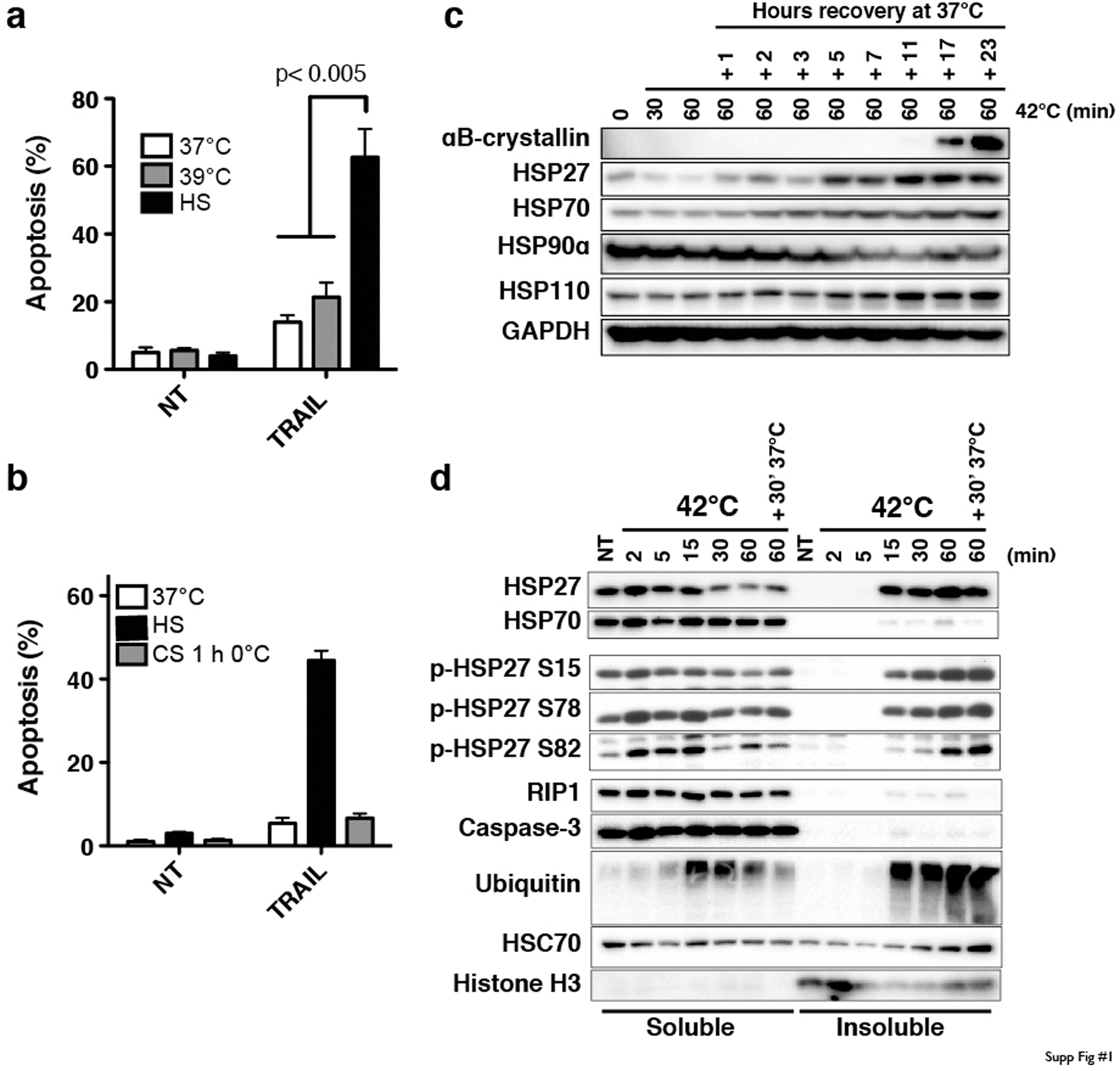

Supplement: Supplementary Material 1 [file cddis201512x2.tif]

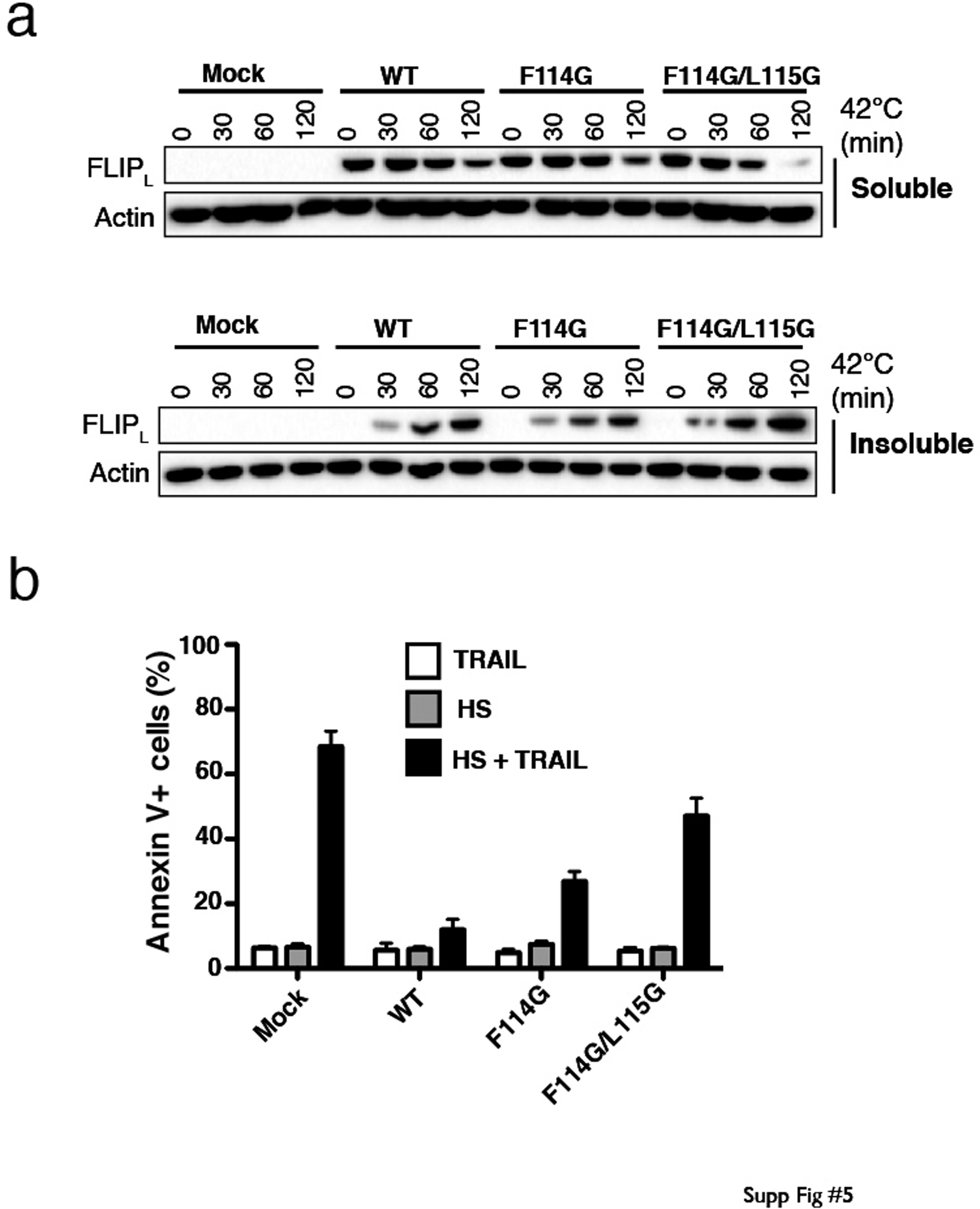

Supplement: Supplementary Material 5 [file cddis201512x6.tif]

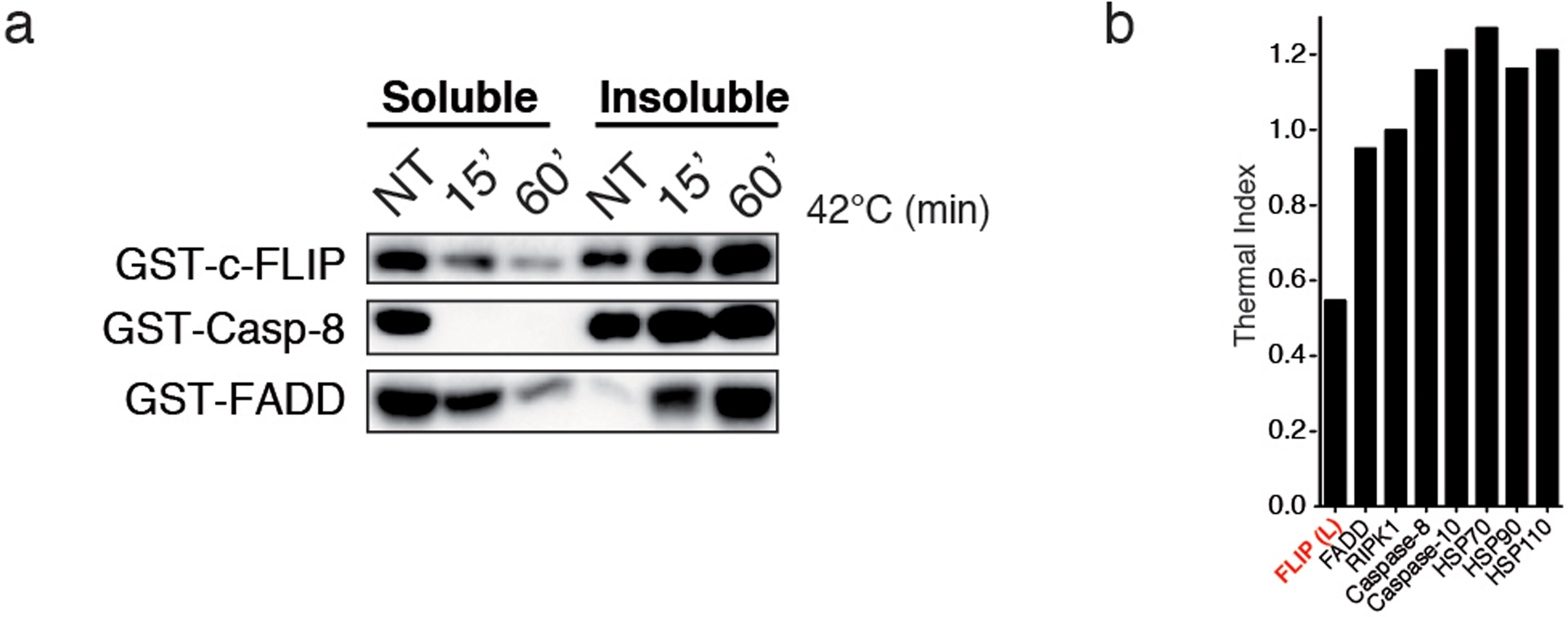

Supplement: Supplementary Material 6 [file cddis201512x7.tif]
